# Impact of atrial fibrillation pattern on outcomes after left atrial appendage closure: lessons from the prospective LAARGE registry

**DOI:** 10.1007/s00392-021-01874-3

**Published:** 2021-05-27

**Authors:** Shinwan Kany, Johannes Brachmann, Thorsten Lewalter, Ibrahim Akin, Horst Sievert, Uwe Zeymer, Jakob Ledwoch, Hüseyin Ince, Dierk Thomas, Matthias Hochadel, Jochen Senges, Paulus Kirchhof, Andreas Rillig

**Affiliations:** 1 Department of Cardiology, University Heart and Vascular Center Hamburg-Eppendorf, Martinistraße 52, 20251 Hamburg, Germany; 2 Department of Cardiology, Angiology and Pneumology, Coburg Hospital, Coburg, Germany; 3 Department of Medicine-Cardiology and Intensive Care, Hospital Munich-Thalkirchen, Munich, Germany; 4 Department of Cardiology, University Hospital Mannheim, Mannheim, Germany; 5 Cardio Vascular Centre, Frankfurt, Frankfurt/Main, Germany; 6 Department of Cardiology, Ludwigshafen Hospital, Ludwigshafen, Germany; 7 Department of Cardiology, Hospital Neuperlach, Munich, Germany; 8 Department of Cardiology, University Hospital Rostock, Rostock, Germany; 9 Department of Cardiology, University Hospital Heidelberg, Heidelberg, Germany; 10 Stiftung Für Herzinfarktforschung (IHF), Ludwigshafen, Germany

**Keywords:** Atrial fibrillation, AF type, Non-paroxysmal AF, Safety outcomes, Left atrial appendage closure

## Abstract

**Background:**

Non-paroxysmal (NPAF) forms of atrial fibrillation (AF) have been reported to be associated with an increased risk for systemic embolism or death.

**Methods:**

Comparison of procedural details and long-term outcomes in patients (pts) with paroxysmal AF (PAF) against controls with NPAF in the prospective, multicentre observational registry of patients undergoing LAAC (LAARGE).

**Results:**

A total of 638 pts (PAF 274 pts, NPAF 364 pts) were enrolled. In both groups, a history of PVI was rare (4.0% vs 1.6%, *p* = 0.066). The total CHA_2_DS_2_-VASc score was lower in the PAF group (4.4 ± 1.5 vs 4.6 ± 1.5, *p* = 0.033), while HAS-BLED score (3.8 ± 1.1 vs 3.9 ± 1.1, *p* = 0.40) was comparable. The rate of successful implantation was equally high (97.4% vs 97.8%, *p* = 0.77). In the three-month echo follow-up, LA thrombi (2.1% vs 7.3%, *p* = 0.12) and peridevice leak > 5 mm (0.0% vs 7.1%, *p* = 0.53) were numerically higher in the NPAF group. Overall, in-hospital complications occurred in 15.0% of the PAF cohort and 10.7% of the NPAF cohort (*p* = 0.12). In the one-year follow-up, unadjusted mortality (8.4% vs 14.0%, *p* = 0.039) and combined outcome of death, stroke and systemic embolism (8.8% vs 15.1%, *p* = 0.022) were significantly higher in the NPAF cohort. After adjusting for CHA_2_DS_2_-VASc and previous bleeding, NPAF was associated with increased death/stroke/systemic embolism (HR 1.67, 95% CI 1.02–2.72, *p* = 0.041).

**Conclusion:**

Atrial fibrillation type did not impair periprocedural safety or in-hospital MACE patients undergoing LAAC. However, after one year, NPAF was associated with higher mortality.

**Graphic abstract:**

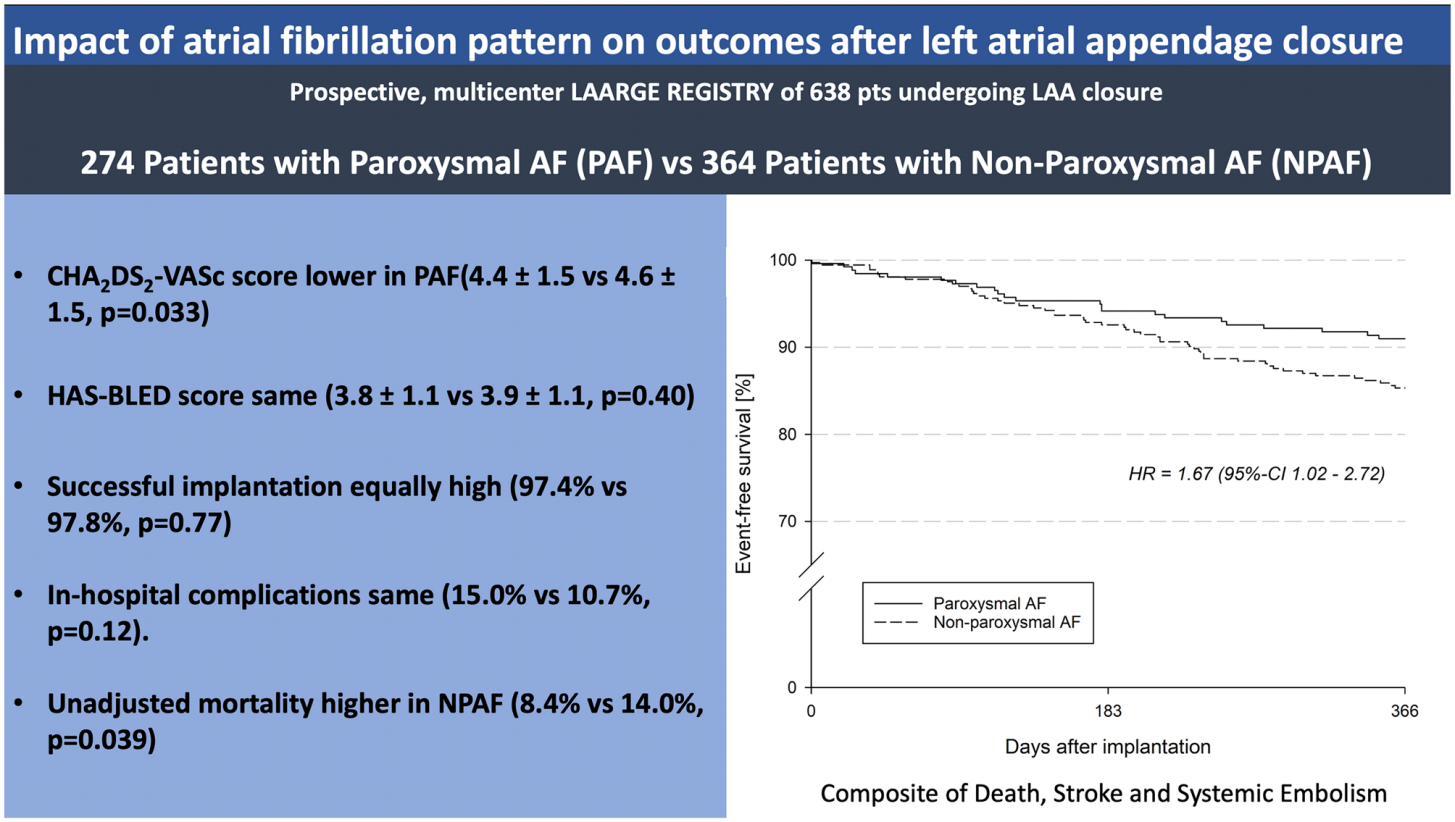

**Supplementary Information:**

The online version contains supplementary material available at 10.1007/s00392-021-01874-3.

## Introduction

Atrial fibrillation (AF) is the most common arrhythmia with more than 33 million people affected worldwide [6]. The risk for stroke is increased fivefold and AF is believed to cause up to 23.5% of all strokes in patients over 80 [22, 25]. AF is a progressive disease with episodes < 7 days referred to as paroxysmal AF (PAF), episodes > 7 days as persistent AF and > 1 year as long-standing persistent AF, while permanent AF is defined as AF without further attempts to restore sinus rhythm according to patient’s and physician’s agreement [15]. Left atrial appendage closure (LAAC) is recommended in patients with contraindications to oral anticoagulation (OAC) and significant stroke risk [26]. The identification of patients who benefit from LAAC still remains challenging. In existing randomized controlled trials, LAAC was non-inferior to OAC in preventing strokes, but associated with significantly less major bleeding [20]. Recently, investigations from several trials and meta-analysis provided evidence that non-paroxysmal AF forms are associated with increased stroke risk and mortality compared with paroxysmal AF, even with anticoagulation [11].

The prospective, real world Left Atrial Appendage Occluder Registry Germany (LAARGE) is a non-randomized and independently funded registry of LAAC in Germany. This study aims to investigate if paroxysmal vs non-paroxysmal forms of AF influence safety and long-term outcomes of LAAC.

## Methods

### Registry structure and data collection

The multicenter LAARGE registry is a prospective, non-randomized study with 38 participating centers. Patients were enrolled from July 2014 to December 2015. The registry is managed by the non-profit organization “Institut für Herzinfarktforschung” (IHF, Ludwigshafen, Germany). There was no funding by industry. Selection of closure devices were left at the operator’s discretion. Written informed consent was obtained from all patients. The privacy measures and data collection have been described previously [3]. Briefly, a web-based electronic case report form was used to collect baseline and procedural data with in-built check for plausibility. IHF conducted the one-year follow-up by reports from the implanting center and via a standardized phone interview. The study was carried out according to the declaration of Helsinki and approved by the ethics committee of the State Chamber of Medicine in Rhineland-Palatinate, Germany (837.173.14 (9412-F), 25.06.2014).

### Procedure and outcomes

The detailed procedural methods have been described previously [9]. Patient selection was conducted according to best medical practice and the most recent guidelines and recommendations. The definitions of paroxysmal AF and non-paroxysmal AF were in accordance with the most recent guidelines [15]. The local implanting center was in charge of procedural protocol, device selection and antithrombotic regime. No limits to device or medication were given by the study protocol. Procedures were carried out in deep sedation using propofol or general anesthesia. Implantation success was defined as a stable position of the device according to the Munich consensus document [24]. Mortality, combined outcome of death and stroke as well as death, stroke and systemic embolism after one year were the primary outcomes. The outcome of death, stroke and systemic embolism was adjusted for CHA_2_DS_2_-VASc and previous bleeding.

### Statistical analysis

Normally distributed continuous data and risk scores are given as means ± standard deviation (SD), otherwise shown as medians with interquartile ranges (25th and 75th percentiles). Categorical data are presented as relative percentages and absolute values. Statistical differences between both groups were compared using either Pearson’s Chi-squared test or Mann–Whitney–Wilcoxon test. Fisher’s exact test was used for rates of in-hospital and follow-up complications. The 12-month event-rates of death, composite outcome of death and stroke, and composite of death, stroke and systemic embolism were calculated by the Kaplan–Meier method. Long-rank test was used to compare the outcomes between the groups. Hazard-ratios (HR) with 95% confidence intervals (CI) were estimated using Cox regression. Adjustment of the composite outcome was done for CHA_2_DS_2_-VASc score and previous bleeding in a multiple Cox regression model and visualized by direct-adjusted survival curves computed in a stratified Cox model, where the expected survival curves are averaged over all patients in the analysis population. All statistical comparisons were two-sided, and *P* values < 0.05 were considered statistically significant. Analyses were performed using the Statistical Analysis System (SAS, Version 9.4, SAS Institute Inc., Cary, NC, USA).

## Results

### Baseline characteristics

A total of 638 patients were included in the analysis. The group with paroxysmal AF (PAF) included 274 patients, while the non-paroxysmal AF (NPAF) group included 364 patients (Table 1). The NPAF group consisted of patients with persistent AF (31.6%) and with longstanding persistent AF or permanent AF (68.4%). Mean age (75.1 ± 8.5 vs 76.6 ± 7.3, *p* = 0.099) and percentage of female patients (43.1% vs 35.7%, *P* = 0.059) were similar. Overall, a history of catheter ablation (CA) with pulmonary vein isolation was rare (4.0% vs 1.6%, *p* = 0.066). The PAF group had significantly less history of congestive heart failure (19.0% vs 33.0%, *p* < 0.001) while the left ventricular ejection fraction (LVEF) (median 60% vs 60%) and the percentage of patients with reduced ejection fraction (LVEF < 40%) were similar. Median heart rate was significantly higher in the NPAF cohort, but still adequately rate controlled.

**Table 1:** Baseline Characteristics of patients with paroxysmal and non-paroxysmal AF

	Paroxysmal AF cohort (*n* = 247)	Non-paroxysmal AF cohort (*n* = 364)	*P* value	Odds ratio (95% CI)
Number of patients	247 (42.9%)	364 (57.1%)		–
Age, years	75.1 ± 8.5	76.6 ± 7.3	0.099	–
Female, %	43.1	35.7	0.059	–
Height, cm	170 (163, 175)	172 (165, 177)	0.014	–
Weight, kg	79 (69, 86)	80 (70, 90)	0.030	–
History of AF				
Paroxysmal AF, %	100.0	0.0		–
Persistent AF, %	0.0	31.6		–
Permanent or LSP AF, %	0.0	68.4		–
History of PVI, %	4.0	1.6	0.066	2.50 (0.91–6.83)
Cardiac history				
Coronary artery disease, %	45.3	46.2	0.82	0.96 (0.70–1.32)
History of MI, %	10.9	9.1	0.43	1.23 (0.73–2.08)
Valvular heart disease, %	20.4	20.9	0.89	0.97 (0.66–1.43)
Cardiomyopathy, %	6.9	7.1	0.92	0.97 (0.52–1.79)
History of congestive heart failure, %	19.0	33.0	< 0.001	0.48 (0.33–0.69)
LVEF, % (median)	60 (50, 60)	60 (50, 60)	0.26	–
LVEF < 40%, %	12.6	11.5	0.65	
Heart rate (median)/min	70 (62, 78)	76 (67, 83)	< 0.001	
Hypertension, %	93.1	92.9	0.92	0.62 (0.43–0.89)
No structural heart disease, %	23.7	17.3	0.045	1.03 (0.56–1.91)
Extracardiac history				
Diabetes mellitus, %	31.8	35.7	0.30	0.84 (0.60–1.17)
Chronic kidney disease, %	33.9	40.7	0.083	0.75 (0.54–1.04)
Vascular disease (e.g., PAD), %	28.1	24.7	0.34	1.19 (0.83–1.70)
Chronic liver disease, %	8.0	11.0	0.21	0.71 (0.41–1.22)
Alcohol use disorder, %	2.6	5.0	0.13	0.51 (0.21–1.23)
Risk scores				
CHA2DS2-VASc Score	4.4 ± 1.5	4.6 ± 1.5	0.033	–
CHA2DS2-VASc Score > 2, %	88.0	91.2	0.18	0.70 (0.42–1.18)
HAS-BLED Score	3.8 ± 1.1	3.9 ± 1.1	0.40	–
Stroke or transient sichemic attack	27.0%	27.2%	0.96	–

Patients with paroxysmal AF have less heart failure and thus lower CHA_2_DS_2_-VASc scores with similar HAS-BLED scores compared with patients with non-paroxsmal AF

*AF* atrial fibrillation, *CI* confidence interval, *LSP* long-standing persistent, *PVI* pulmonary vein isolation, *MI* myocardial infarction, *LVEF* left ventricular ejection fraction, *PAD* peripheral artery disease; displayed are percentages and numbers or median and quartiles; *P* values < 0.05 are considered significant, tested with either Pearson’s chi-squared test or Mann–Whitney–Wilcoxon test

The CHA_2_DS_2_-VASc score was lower in the PAF group (4.4 ± 1.5 vs 4.6 ± 1.5, *p* = 0.033) but the percentage of patients with a CHA_2_DS_2_-VASc score > 2 was statistically similar (88.0% vs 91.2%, *p* = 0.18). There was no difference in the HAS-BLED score (3.8 ± 1.1 vs 3.9 ± 1.1, *p* = 0.40). An overview of indications for LAAC is given in the supplemental Table 1.

### Left atrial appendage anatomy and procedural data

The LA diameter (median 46 mm vs 49 mm, *p* < 0.001) and LA volume (20.5 cm^2^ vs 29.0 cm^2^, *p* = 0.001) were significantly smaller in the PAF cohort compared to the NPAF cohort (supplemental Table 2). The prevalence of LAA thrombus (0.4% vs 0.9%, *p* = 0.50) and LAA sludge (14.1% vs 15.3%, *p* = 0.70) was similar in both groups. Acute implantation success of LAAC devices was equally high (97.4% vs 97.8%, *p* = 0.77) in both groups (Table 2). The PAF cohort presented significantly more often in sinus rhythm (69.0% vs 2.2%, *p* < 0.001) and significantly less in AF (23.7% vs 92.9%, *p* < 0.001). Most procedures were done under conscious sedation (85.0% vs 83.5%, *p* = 0.62), followed by general anesthesia (10.6% vs 12.1%, *p* = 0.57). There was no difference in device selection. The number of device-retractions and repositioning (1.6 ± 1.3 vs 1.7 ± 1.2, *p* = 0.069) was comparable in both groups but skewing towards the NPAF group. The PAF cohort had significantly longer procedural (61 min vs 55 min, *p* = 0.004) and fluoroscopy (11 min vs 9 min, *p* = 0.002) times. There was no significant difference in peridevice leak (4.6% vs 5.6%, *p* = 0.57) or left-to-right shunts (6.7% vs 4.5%, *p* = 0.22) after the procedure.

**Table 2:** Procedural data

	Paroxysmal AF cohort (*n* = 274)	Non-paroxysmal AF cohort (*n* = 364)	*P* value	Odds ratio (95% CI)
Implant success, %	97.4	97.8	0.77	0.86 (0.31–2.39)
Rhythm at implant				
Sinus rhythm, %	69.0	2.2	< 0.001	98.95 (46.93–208.63)
Atrial fibrillation, %	23.7	92.9	< 0.001	0.02 (0.01–0.04)
Pacing, %	8.4	5.8	0.20	1.50 (0.81–2.76)
Anesthesia				
Conscious sedation, %	85.0	83.5	0.62	1.12 (0.72–1.72)
General anesthesia, %	10.6	12.1	0.57	0.86 (0.53–1.42)
LAAC device				
Watchman, %	45.3	42.4	0.48	1.12 (0.82–1.54)
Amplatzer Cardiac Plug, %	26.3	28.9	0.46	0.88 (0.62–1.25)
Amplatzer Amulet, %	25.5	25.6	0.98	1.00 (0.70–1.43)
Other device*, %	2.9	3.0	0.94	0.96 (0.38–2.43)
Periprocedural data				
Sheath retractions	1.6 ± 1.3, *N* = 263	1.7 ± 1.2, *N* = 356	0.069	–
Duration, min	61 (46, 85)	55 (42, 74)	0.004	–
Fluroroscopy duration, min	11 (8, 16)	9 (7, 14)	0.002	–
Dose area product, cGy*cm^2^	1999 (851, 4010)	2091 (856, 4512)	0.84	–
Device dislodgment, %	1.8 (5)	1.1 (4)	0.44	1.67 (0.44–6.27)
Catheter-based retrieval, %	5/5	4/4		–-
Surgical retrieval, %	0/5	0/4		–-
Peridevice leak, %	4.6 (12)	5.6 (20)	0.57	0.81 (0.39–1.68)
< 3 mm	8/12	16/20		0.50 (0.10–2.54)
3—5 mm	4/12	4/20		2.00 (0.39–10.16)
> 5 mm	0/12	0/20		–-
Left–right shunt, %	6.7	4.5	0.22	1.53 (0.77–3.06)

*AF* atrial fibrillation, *CI* confidence interval, *LAAC* left atrial appendage closure; displayed are percentages and numbers or median and quartiles

*Other devices include Occlutech, LAmbre and LARIAT; *P* values < 0.05 are considered significant, tested with Fisher`s exact test

### In-hospital safety data

Incidences of MACCE (Death, Stroke or MI) were equally rare (0.7% vs 0.3%, *p* = 0.58) in both groups (Table 3). Other severe complications (4.7% vs 3.6%, *p* = 0.55), including severe bleeding and AV-Fistula, were similar. Moderate complications were similar in both groups (10.9% vs 8.8%, *p* = 0.42) as well as minor complications (2.9% vs 2.5%, *p* = 0.81). The overall incidence of combined severe and moderate complications was numerically higher in the PAF cohort (15.0% vs 10.7%, *p* = 0.12).

**Table 3:** In-hospital safety data after the procedure

	Paroxysmal AF cohort (*n* = 274)	Non-paroxysmal AF cohort (*n* = 364)	*P* value	Odds ratio (95% CI)
MACCE (death, MI, stroke), %	0.7	0.3	0.58	
Death, %	0.7	0.0	0.18	–
MI, %	0.0	0.3	1.00	–
Stroke, %	0.0	0.3	1.00	–
Other severe complications, %	4.7	3.6	0.55	1.34 (0.61–2.95)
Severe bleeding, %	1.1	1.1	1.00	1.00 (0.22–4.49)
AV-Fistula/Aneurysmal hematoma, %	1.1	0.8	1.00	1.33 (0.27–6.65)
Pericardial effusion—surgical treatment, %	0.7	0.0	0.18	–
Pericardial effusion—interventional treatment, %	2.2	1.9	1.00	1.14 (0.38–3.44)
Hemo-/pneumothorax—surgical treatment, %	0.0	0.0	–	–
Device dislodgment—surgical treatment, %	0.0	0.0	–	–
Device dislodgment—interventional treatment, %	0.4	0.3	1.00	1.33 (0.08–21.29)
MACCE + other severe complication, %	5.5	3.8	0.34	1.45 (0.69–3.05)
Moderate complications, %	10.9	8.8	0.42	1.28 (0.75–2.16)
TIA, %	0.0	0.0	–	–
Non-fatal CPR, %	0.4	0.5	1.00	0.66 (0.06–7.35)
Moderate bleeding, %	1.8	1.9	1.00	0.95 (0.30–3.02)
Access site infection, %	0.4	0.0	0.43	–-
Groin hematoma, %	2.2	3.3	0.48	0.66 (0.24–1.77)
Pericardial effusion—conservative treatment, %	2.9	0.8	0.063	3.62 (0.95–13.77)
Hemo-/pneumothorax—interventional treatment, %	0.4	0.3	1.00	1.33 (0.08–21.35)
Hemo-/pneumothorax—conservative treatment, %	0.0	0.0	–	–
Device dislodgment—at index procedure, %	1.5	0.8	0.47	1.78 (0.39–8.01)
Minor complications, %	2.9	2.5	0.81	1.19 (0.45–3.12)
Overall complications (severe and moderate), %	15.0	10.7	0.12	1.47 (0.92–2.35)

The AF form does influence safety measures

*AF* atrial fibrillation, *MI* myocardial infarction, *AV* arteriovenous, *TIA* transient ischemic attack, *CPR* cardiopulmonary resuscitation; displayed are percentages and numbers; *P* values < 0.05 are considered significant, tested with tested with Fisher’s exact test

### Antithrombotic therapy

Before the procedure, therapeutic anticoagulation was comparable in both groups (47.1% vs 50.8%, *p* = 0.35). Single antiplatelet therapy (SAPT) was significantly higher in the PAF cohort compared with the NPAF cohort (21.2% vs 11.8%, *p* = 0.001). At discharge, dual antiplatelet therapy (DAPT) was the main antithrombotic therapy in both groups (83.1% vs 85.2%, *p* = 0.48). There were no significant differences in the antithrombotic therapy at discharge or at the one-year follow-up (details in Table 4).

**Table 4:** Antithrombotic therapy

	Paroxysmal AF cohort	Non-paroxysmal AF cohort	*P* value	Odds ratio (95% CI)
Therapy at admission	*n* = 274	*n* = 364		
Anticoagulation, %	47.1	50.8	0.35	0.86 (0.63–1.18)
DAPT, %	7.7	6.9	0.70	1.13 (0.62–2.06)
SAPT, %	21.2	11.8	0.001	2.00 (1.30–3.08)
Double antithrombotic therapy, %	8.8	8.8	0.99	1.00 (0.57–1.73)
Triple antithrombotic therapy, %	2.2	3.6	0.31	0.60 (0.23–1.61)
No antithrombotic therapy, %	13.1	18.1	0.088	0.68 (0.44–1.06)
Therapy at discharge, %	*n* = 272	*n* = 364		
Anticoagulation, %	3.3	2.5	0.53	1.35 (0.53–3.45)
DAPT, %	83.1	85.2	0.48	0.86 (0.56–1.31)
SAPT, %	4.0	1.9	0.11	2.15 (0.82–5.62)
Double antithrombotic therapy, %	7.0	6.9	0.95	1.02 (0.55–1.89)
Triple antithrombotic therapy, %	2.2	2.7	0.67	0.80 (0.29–2.22)
No antithrombotic therapy, %	0.4	0.5	0.74	0.67 (0.06–7.40)
PPI, %	48.0	46.3	0.67	1.07 (0.78–1.47)
NSAID, %	8.5	6.1	0.24	1.44 (0.78–2.64)
Therapy at one-year follow-up	*n* = 223	*n* = 284		
Anticoagulation, %	5.4	4.2	0.54	1.29 (0.57–2.93)
DAPT, %	6.3	6.7	0.85	0.93 (0.46–1.91)
SAPT, %	78.9	73.6	0.16	1.34 (0.89–2.04)
Double antithrombotic therapy, %	0.9	1.8	0.41	0.50 (0.10–2.63)
Triple antithrombotic therapy, %	0.0	0.7	0.21	–
No antithrombotic therapy, %	8.5	13.0	0.11	0.62 (0.35–1.11)
PPI, %	43.5	39.4	0.36	1.18 (0.83–1.69)
NSAID, %	5.4	4.9	0.82	1.10 (0.50–2.42)

*OR* odds ratio, *CI* confidence interval, *DAPT* dual antiplatelet therapy, *SAPT* single antiplatelet therapy, *PPI* proton pump inhibitor, *NSAID* non-steroidal anti-inflammatory drugs; displayed are percentages and numbers; *P* values < 0.05 are considered significant, tested with either Pearson’s chi-squared test or Mann–Whitney–Wilcoxon test

### Follow-up safety data

**Fig. 1:**
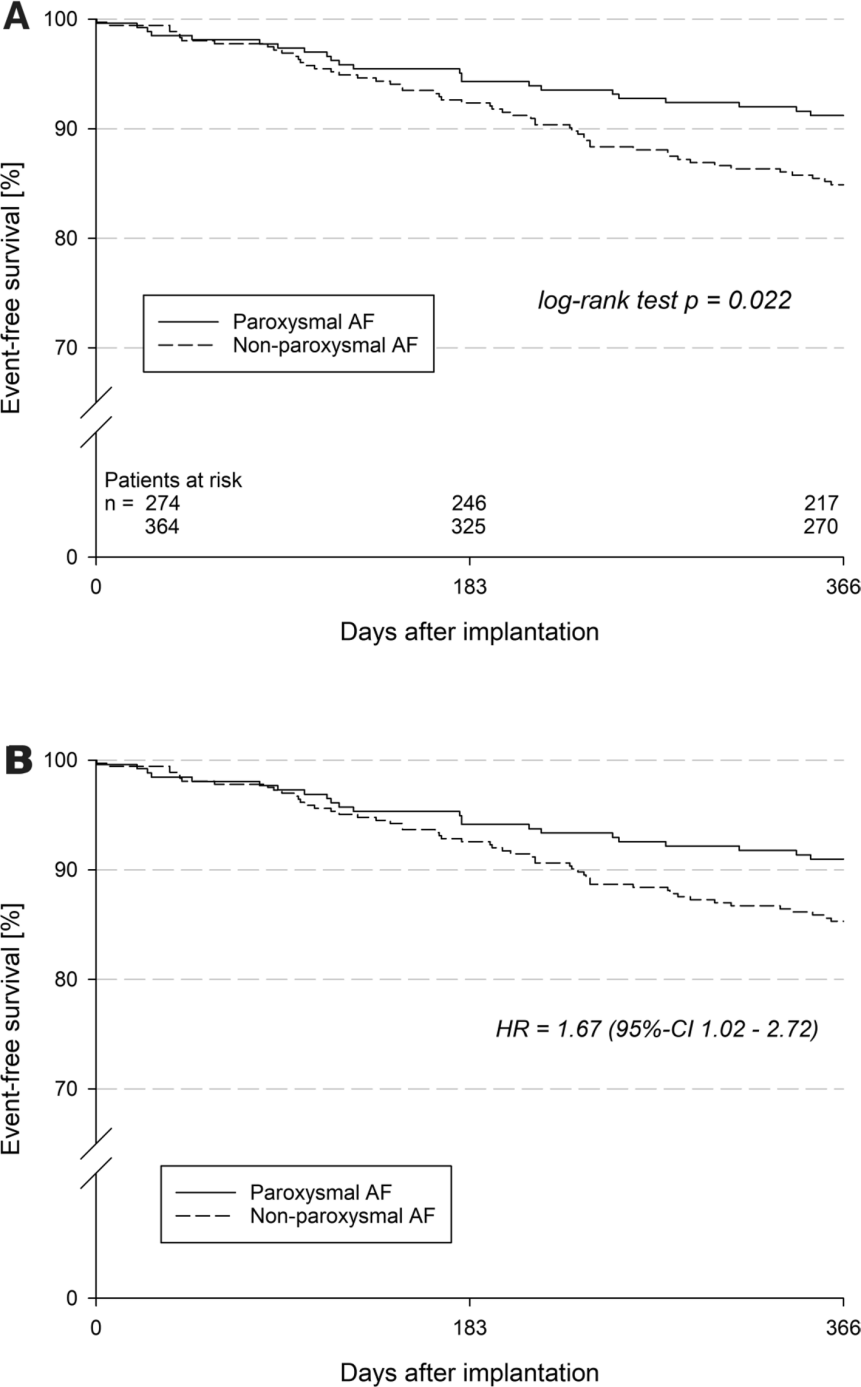
One-year outcomes of event-free survival (death/stroke/systemic embolism) unadjusted **a** and adjusted for for CHA_2_DS_2_-VASc and previous bleeding **b** in patients with paroxysmal AF or non-paroxysmal AF; *P* values < 0.05 are considered significant, tested with either Pearson’s chi-squared test or Mann–Whitney–Wilcoxon test

In the echocardiographic FU after ca. 100 days (103d (47d, 194d) vs 97d (54d, 186d)), LA thrombi (2.1% vs 7.3%, *p* = 0.12) and peridevice leak > 5 mm (0.0% vs 7.1%, *p* = 0.53) were numerically higher in the NPAF group. Overall device dislodgment was rare (2.2% vs 2.5%, *p* = 1.00). Groin complications (2.6% vs 3.9%, *p* = 0.50) were rare in both cohorts. Pericardial effusion was numerically higher in the PAF cohort (6.2% vs 3.3%, *p* = 0.09, OR 1.94 (0.91–4.14)). There was no difference in stroke (0.4% vs 1.4%, *p* = 0.24) or TIA (0.4% vs 0.3%, *p* = 1.0), however, MI occurred significantly less often (0.0% vs 1.7%, *p* = 0.04) in the PAF cohort compared with the NPAF cohort. In the unadjusted outcomes, the PAF cohort demonstrated significantly lower all-cause mortality (8.4% vs 14.0%, *p* = 0.039), combined death and stroke (8.8% vs 14.8%, *p* = 0.028) and combined outcome of death, stroke and systemic embolism (8.8% vs 15.1%, *p* = 0.022) (Table 5). In the adjusted (CHA_2_DS_2_-VASc and previous bleeding) outcome of event-free survival NPAF was associated with increased composite outcome of death/stroke/systemic embolism (HR 1.67, 95% CI 1.02–2.72, *p* = 0.041) (Fig. 1).

**Table 5:** Follow-up safety data

	Paroxysmal AF (*n* = 274)	Non-paroxysmal AF (*n* = 364)	*P* value	Odds ratio (95% CI)
Echocardiography FU documented, %	34.9 (95/272)	34.1 (124/364)	0.87	–
Days to echo FU	103 (47, 194)	97 (54, 186)	0.94	–
Peridevice leak, %	16.8 (16/95)	22.6 (28/124)	0.31	0.69 (0.35–1.37)
< 3 mm	13/16	22/28	1.00	1.18 (0.25–5.55)
3—5 mm	3/16	4/28	0.69	1.38 (0.27–7.15)
> 5 mm	0/16	2/28	0.53	0.28 (0.06–1.31)
LA thrombus, %	2.1 (2/94)	7.3 (9/123)	0.12	0.28 (0.06–1.31)
LA sludge, %	0.0 (0/27)	2.7 (1/37)	1.00	–
One year follow-up				
One-year FU documented, %	97.1 (266/274)	98.1 (357/364)	0.44	–
Days to one-year FU	377 (367, 402)	380 (367, 411)	0.50	–
Device dislodgment, %	2.2 (6)	2.5 (9)	1.00	0.88 (0.31–2.51)
Surgical treatment	0/6	3/9	0.23	–
Interventional treatment	6/6	4/9	0.044	–
Conservative treatment	0/6	2/9	0.49	–
Groin complications, %	2.6 (7)	3.9 (14)	0.50	0.66 (0.26–1.65)
Surgical treatment	1/7	2/14	1.00	1.00 (0.07–13.37)
Blood transfusion	0/7	0/14	–	–
Conservative treatment	6/7	12/14	1.00	1.00 (0.07–13.37)
Pericardial effusion, %	6.2 (16)	3.3 (12)	0.087	1.94 (0.91–4.14)
Surgical treatment	2/16	0/12	0.49	–
Interventional treatment	7/16	7/12	0.70	0.56 (0.12–2.53)
Conservative treatment	7/16	5/12	1.00	1.09 (0.24–4.95)
Stroke, %	0.4	1.4	0.24	0.31 (0.03–2.81)
TIA, %	0.4	0.3	1.00	0.26 (0.03–2.27)
MI, %	0.0	1.7	0.040	1.33 (0.08–21.37)
Bleeding (severe or moderate), %	7.3	6.9% (25)	0.88	–
Severe bleeding, %	1.5	2.8	0.41	1.07 (0.58–1.97)
Moderate bleeding, %	5.9	4.1	0.35	0.52 (0.16–1.69)
Composite outcomes†				
Mortality, %	8.4	14.0	0.039	0.59 (0.36–0.98) HR
Death/stroke, %	8.8	14.8	0.028	0.58 (0.36–0.95) HR
Death/stroke/SE, %	8.8	15.1	0.022	0.57 (0.35–0.93) HR

*OR* odds ratio, *CI* confidence interval, *TIA* transient ischemic attack, *MI* myocardial infarction, *SE* systemic embolism, *KM* Kaplan–Meier estimate, *LO* Log-rank test, *HR* hazard ratio; displayed are percentages and numbers; *P* values < 0.05 are considered significant, tested with either Fisher’s exact test or Mann–Whitney–Wilcoxon test

^**†**^Kaplan–Meier estimates at 1 year after the index procedure, compared by log-rank test

## Discussion

Among patients undergoing LAAC, non-paroxysmal forms of AF are associated with higher rates of death, stroke or systemic embolism after one year in a prospective real-world setting regardless of the CHA_2_DS_2_-VASc score. Complications are comparable between patients with paroxysmal or non-paroxysmal AF, though procedure and fluoroscopy times are longer in the former.

Recently, a case series showed that LAAC is associated with acute brain lesion in MRI of unknown clinical relevance [21]. The impact of AF type on stroke risk has long been debated. A *post-hoc* analysis of the ACTIVE-W trial found no association of AF type on thromboembolic events. However, differences in anticoagulation between PAF and persistent AF and low CHADS_2_ scores were a major limitation [16]. In a *post-hoc* analysis of the ROCKET-AF trial, persistent AF was significantly associated with higher systemic embolism, stroke and all-cause mortality [23]. Recently, a meta-analysis of 12 studies, with almost 100.000 patient showed that NPAF was associated with a higher risk for thromboembolism and all-cause mortality, no difference in bleeding was observed [11]. Interestingly, sub-analyses of the ENTRUST-AF and ENSURE-AF trials showed an association of MI and PAF not observed in patients with persistent AF [13, 14]. The authors propose that PAF is associated with microcirculatory flow abnormalities and may increase events in vulnerable myocardium [13, 14]. However, several limitations may limit the generalization of the findings. For Instance, patients with PAF in ENSURE-AF were significantly more likely to be without anticoagulation at baseline compared with NPAF patients (47% vs 23%, *p* < 0.0001) and follow-up encompassed only 58 days [13, 14]. While the increased stroke risk and mortality for NPAF has been shown repeatedly, the influence of AF types on LAA thrombus formation and outcomes after LAAC has not been a focus of research [5].

Our cohort had comparable CHA_2_DS_2_-VASc scores (PAF: 4.4 ± 1.5 vs NPAF: 4.6 ± 1.5) to other registries such as the European EWOLUTION registry (4.5 ± 1.6). While the one-year mortality in EWOLUTION was 9.8% and thus comparable to the PAF cohort (8.4%), the NPAF cohort demonstrated a higher mortality of 14% [2]. Incidences of major bleeding as a surrogate for differences in major comorbidities were similar in EWOLUTION (2.6%) and our cohort (2.8%). Furthermore, CA and antiarrhythmic drug therapy have been reported to potentially decrease stroke risk and mortality in select patients [10, 17]. Our study shows that the cohort undergoing LAAC is treated very rarely with CA (4.0% vs 1.6%). The influence of CA on the prevention of stroke in AF is still studied and may be of importance in the population undergoing LAAC [4, 19]. We also report a higher incidence of congestive heart failure in NPAF patients compared with PAF despite similar LVEF. One aspect that may serve as an explanation may be the rate control of atrial fibrillation. While we did observe a higher median heart rate with NPAF compared with PAF, the overall heart rate was still sufficiently rate controlled (median 76/min (67–83)). Certainly, heart failure with preserved ejection fraction may be a factor, considering the NPAF cohort were much more likely in AF than compared with PAF patients.

What is more, there is evidence that NPAF is associated with larger LAA volume as well as non-chicken-wing LAA morphologies [18]. These morphologies are associated with a higher risk for stroke [7]. This is furthermore supported by a recent work showing that long standing persistent AF leads to larger LAA sizes, which require larger sizes of LAAC devices and lead to more residual leaks after closure [12]. Considering that most patients in the NPAF cohort were diagnosed with long-standing persistent or permanent AF, it is highly likely they have a longer history of AF compared with PAF patients.

While we could observe increasing volume of LA and LAA ostia in the NPAF cohort, this was not associated with more residual leaks in the follow-up. We observed longer fluoroscopy and procedure times in PAF patients, which might be explained by the higher rate of sinus rhythm during implant and thus potentially more challenging LAA movement.

Our data shows numerically higher incidences of LA thrombus in the NPAF cohort. In an analysis of 1739 patients in prospective trials or registries in the US receiving a Watchman device, DRT was significantly associated with large LAA diameter and permanent atrial fibrillation and higher risk of stroke [8]. Likewise, in the prospective Amulet Observational study the incidence of DRT was reported with 1.7% with larger LAA orifice being a significant predictor for DRT and associated increased risk of stroke [1]. In line with these observations, in our cohort NPAF was associated with an increased risk for death, stroke or systemic embolism. Our observed findings remain after adjusting for CHA_2_DS_2_-VASc score, underlining the effect NPAF may have on outcomes after LAAC. Future studies are needed and, ultimately, randomized trials to evaluate different antithrombotic strategies after LAAC depending on AF patterns observed.

## Limitations

Confounding factors cannot be excluded due to the observational registry format. Additionally, there was no standardized process for patient screening, device selection and LAAC procedure.

There may have been significant differences between the operators’ experience as well as the center volume of procedures. Also, increased experience and technical improvements may lead to more favorable outcomes in a contemporary study. Newer devices such as the Watchman FLX were not included in this study. Furthermore, we lack sufficient follow-up echocardiography data to report three-month and one-year incidences of DRT and peri-device leak. Centers were encouraged to enroll patients consecutively but not obligated; therefore, a selection bias cannot be excluded. There was no monitoring of AF burden or changes of AF pattern during follow-up but since most patients in the NPAF cohort were in longstanding persistent or permanent AF, the AF burden is unlikely to have changed during follow-up.

Centers were also encouraged to report adverse events which may include a certain reporting bias.

## Conclusion

Patients with non-paroxysmal forms of atrial fibrillation undergoing LAAC are more likely to have larger LA volume as well as larger LAA size and orifices. This did, however, not impair periprocedural safety or in-hospital MACE. After one year, NPAF was associated with higher mortality, stroke or systemic embolism.

## Supplementary Information

Below is the link to the electronic supplementary material.

Supplementary file1 (DOCX 49 KB)

## Data Availability

All data and material available at IHF Ludwigshafen.

## Abbreviations

AF: Atrial fibrillation
CA: Catheter ablation
DRT: Device-related thrombus
LAA: Left atrial appendage
LAAC: Left atrial appendage closure
NPAF: Non-paroxysmal atrial fibrillation
OAC: Oral anticoagulation
PAF: Paroxysmal atrial fibrillation
