# Supplementary material for: Impact of atrial fibrillation pattern on outcomes after left atrial appendage closure: lessons from the prospective LAARGE registry
Source: Clin Res Cardiol. 2021 May 27;111(5):511–21. doi: 10.1007/s00392-021-01874-3 (PMC9054864; doi:10.1007/s00392-021-01874-3)
Supplement: Supplementary file 1 — Supplementary file1 (DOCX 49 KB) [file 392_2021_1874_MOESM1_ESM.docx]

SUPPLEMENT

**Supplemental table 1: Indications for left atrial appendage closure:** CVI: cerebrovascular insult; OAC: oral anticoagulation; TIA: transient ischemic attack; INR: international normalized ratio; displayed are percentages and numbers; P-values <0.05 are considered significant, tested with either Pearson chi-squared test or Mann-Whitney-Wilcoxon test

|  | **Paroxysmal AF** | **Non-paroxysmal AF** | **P-value** |
| --- | --- | --- | --- |
| History of bleeding | 79.9 % (219/274) | 79.1 % (288/364) | 0.80 |
| **Severity of bleeding** |  |  | 0.23 |
| - severe | 53.9 % (118/219) | 46.5 % (134/288) |  |
| - moderate | 33.3 % (73/219) | 42.4 % (122/288) |  |
| - mild | 12.8 % (28/219) | 11.1 % (32/288) |  |
| **History of CVI** | 27.0 % (74) | 27.2 % (99) | 0.96 |
| - History of stroke | 21.2 % (58) | 21.7 % (79) | 0.87 |
| - History of TIA | 9.1 % (25) | 7.4 % (27) | 0.44 |
| Contraindication to OAC | 20.8 % (57) | 17.6 % (64) | 0.30 |
| Labile INR | 8.4 % (23) | 8.5 % (31) | 0.96 |
| Poor adherence to OAC | 4.7 % (13) | 5.5 % (20) | 0.67 |
| Patient wish | 27.4 % (75) | 23.6 % (86) | 0.28 |
| Other indication | 6.9 % (19) | 10.7 % (39) | 0.10 |

**Supplemental table 2: Left atrial appendage anatomy before the procedure:** Patients with PAF have smaller LA sizes and LAA orifices. AF: atrial fibrillation; CI: confidence interval; LA: left atrium; LAA: left atrial appendage; displayed are percentages and numbers or median and quartiles; P-values <0.05 are considered significant, tested with either Pearson chi-squared test or Mann-Whitney-Wilcoxon test

|  | **Paroxysmal AF cohort**  **(n=247)** | **Non-paroxysmal AF cohort (n=364)** | **P-value** | **Odds ratio (95% - CI)** |
| --- | --- | --- | --- | --- |
| LA diameter, mm | 46 (42, 50) | 49 (45, 53) | < 0.001 | --- |
| LA area, cm^2^ | 20.5 (16.0, 29.5) | 29.0 (26.0, 35.0) | 0.001 | --- |
| **LAA thrombus formation** |  | | | |
| - LAA thrombus, % | 0.4 | 0.9 | 0.50 | 0.47 (0.05-4.54) |
| - LAA sludge, % | 14.1 | 15.3 | 0.70 | 0.91 (0.57-1.45) |
| **LAA morphology** |  | | | |
| - Cactus, % | 8.5 | 9.6 | 0.65 | 0.88 (0.49-1.57) |
| - Chicken wing, % | 41.9 | 46.7 | 0.26 | 0.83 (0.59-1.15) |
| - Windsock, % | 14.8 | 15.7 | 0.79 | 0.94 (0.59-1.49) |
| - Cauliflower, % | 17.4 | 14.2 | 0.30 | 1.27 (0.81-2.00) |
| - Atypical, % | 17.4 | 13.9 | 0.26 | 1.30 (0.83-2.05) |
| **No. of lobi** |  | | | |
| - 1 Lobus, % | 56.8 | 52.2 |  | --- |
| - 2 Lobi, % | 34.2 | 42.3 |  | --- |
| - > 2 Lobi, % | 9.0 | 5.5 |  | --- |
| **LAA dimensions** |  |  |  | --- |
| Dimension ostium measured (at least 1 plane), % | 84.0 | 84.9 | 0.76 | 0.93 (0.59-1.47) |
| - Ostium 0°, mm | 19.0 (17.0, 22.0) | 20.5 (18.0, 22.0) | 0.010 | --- |
| - Ostium 45°, mm | 19.0 (17.0, 22.0) | 20.0 (18.0, 23.0) | < 0.001 | --- |
| - Ostium 90°, mm | 19.0 (16.0, 21.0) | 21.0 (18.0, 23.0) | < 0.001 | --- |
| - Ostium 135°, mm | 20.0 (17.0, 23.0) | 20.0 (18.0, 22.0) | 0.11 | --- |
